# Supplementary material for: Abolishing Retro-Transduction of Producer Cells in Lentiviral Vector Manufacturing
Source: Viruses. 2024 Jul 29;16(8):1216. doi: 10.3390/v16081216 (PMC11359676; doi:10.3390/v16081216)
Supplement: Supplementary file 1 [file viruses-16-01216-s001.zip › Figures_Supplementary/Supplementary Table 1.pdf]

Primer name	Primer sequence	Objective
U3 FW	GGAAGGGCTAATTCACTCCCA	Transduction-in-Production (TIPs) detection, FW
MH532 RV	GAGTCCTGCGTCGAGAGAGC	Transduction-in-Production (TIPs) detection, RV
Endo sLDLR RV	GCTTGGTGAGACATTGTCACT	Amplification of endogenous cellular LDLR by RT-qPCR
Exo sLDLR RV	ACTGCATCCATGGCAGCC	Amplification of exogenous cellular LDLR by RT-qPCR
Exo sLDLR RV	TGGATGTCCCTGCTGATGACG	Amplification of exogenous cellular LDLR by RT-qPCR
Total sLDLR FW	GCAAATCCGGGGACTTCA	Amplification of total LDLR by RT-qPCR
Total sLDLR RV	TCCTGGGAGCACGTCTT	Amplification of total LDLR by RT-qPCR
Kozak-RAP FW	AGCACGTGAGATCTGAATTGCCACCATG GCGCCGCGGAGGGTC	Introduce RAP in pMD2 vector
Kozak-RAP RV	AAGTTAGTAGCTCCGCTTCCGCGTGCTC TGGAGATCCTGCCG	Introduce RAP in pMD2 vector
Kozak RAP Gluc FW	AGCACGTGAGATCTGAATTGCCACCATG GGTGTGAAAGTTCTCTTCGCGCTTATTTG TATCGCGGTGGCTGAGGTCACCGGAGC GCCGCGGAGGGTCAGGTC	Introduce GLUC signal peptide before RAP in pMD2 vector
RAP H257F+H259F+Y260C FW	TCGAAGCCAAAATCGAGAAGTTCAACTT CTGCCAGAAGCAGCTGGAGATTGCGCA C	Introduce three mutations (H257F, H259F, Y260C) in RAP
RAP H257F+H259F+Y260C RV	GTGCGCAATCTCCAGCTGCTTCTGGCA GAAGTTGAACTTCTCGATTTTGGCTTCGA	Introduce three mutations (H257F, H259F, Y260C) in RAP
RAP H268F FW	CAGAAGCAGCTGGAGATTGCGTTCGAGA AGCTGAGGCACGCAGAGAG	Introduce a mutation (H268F) in RAP
RAP H268F RV	CTCTCTGCGTGCCTCAGCTTCTCGAACG CAATCTCCAGCTGCTTCTG	Introduce a mutation (H268F) in RAP
RAP H290F + T297C FW	TGAGCCGCAGCCGCGAGAAGTTCGCC CTGCTGGAGGGGCGGTGCAAGGAGCT GGGCTACACGGTG	Introduce the mutations (H290F, T297C) in RAP
RAP H290F + T297C RV	CACCGTGTAGCCCAGCTCCTTGCACCG CCCCTCCAGCAGGGCGAACTTCTCGC GGCTGCGGCTCA	Introduce the mutations (H290F, T297C) in RAP
LDLR guide RNA 1	GATGAACCCATCAAAGAGTG	Guide RNA to delete a region in LDLR gene
LDLR guide RNA 2	GCCATCGCAGACCCACTTGT	Guide RNA to delete a region in LDLR gene
gD LDLR FW	GTTCCTTCTTTGTGTCCTCCA	LDLR KO confirmation FW
gD LDLR RV	ACAGGGTGGGCAGAGTGGAGT	LDLR KO confirmation RV
