# Supplementary figures and images for: Abolishing Retro-Transduction of Producer Cells in Lentiviral Vector Manufacturing
Source: Viruses. 2024 Jul 29;16(8):1216. doi: 10.3390/v16081216 (PMC11359676; doi:10.3390/v16081216)

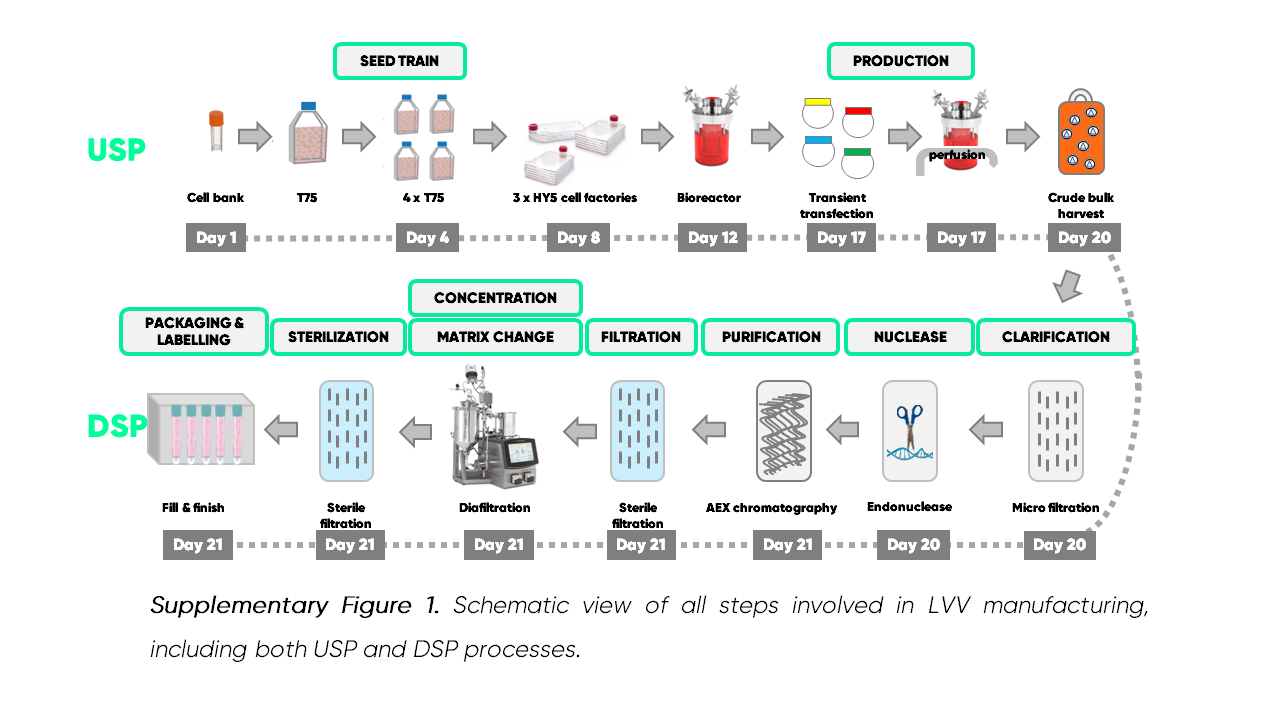

Supplement: Supplementary file 1 [file viruses-16-01216-s001.zip › Figures_Supplementary/fig_supp_1.PNG]

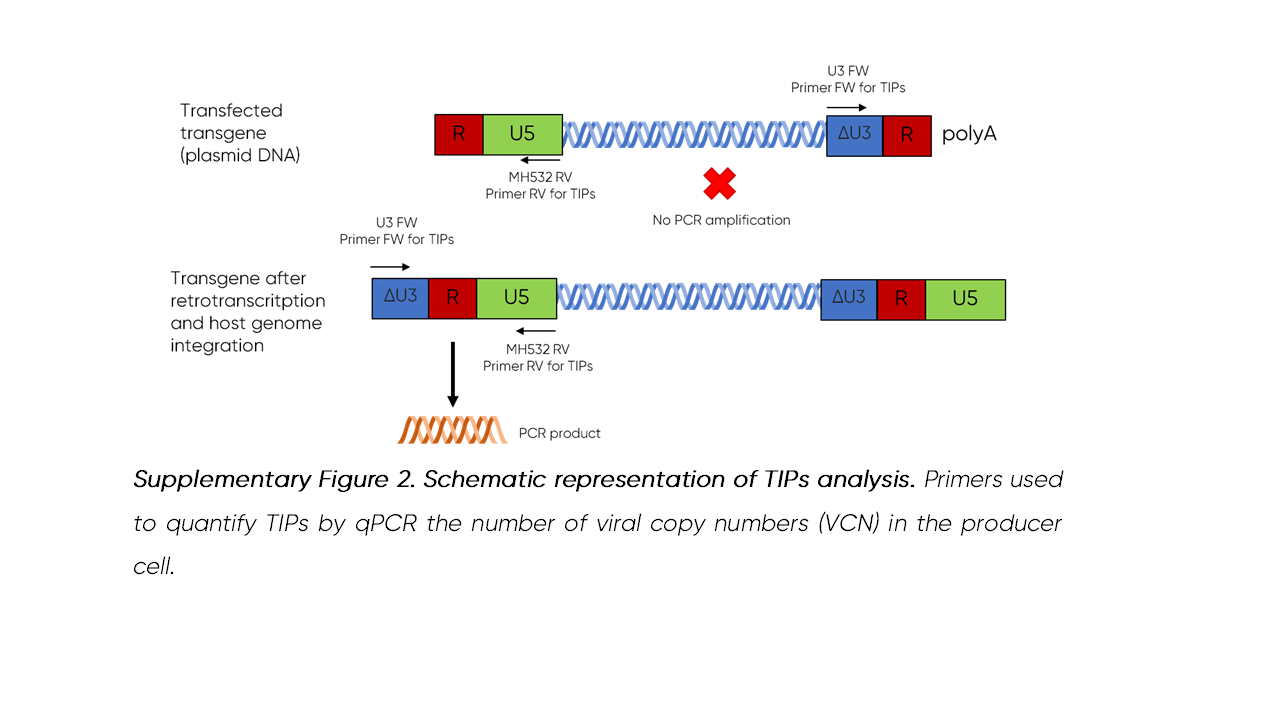

Supplement: Supplementary file 1 [file viruses-16-01216-s001.zip › Figures_Supplementary/fig_supp_2.PNG]

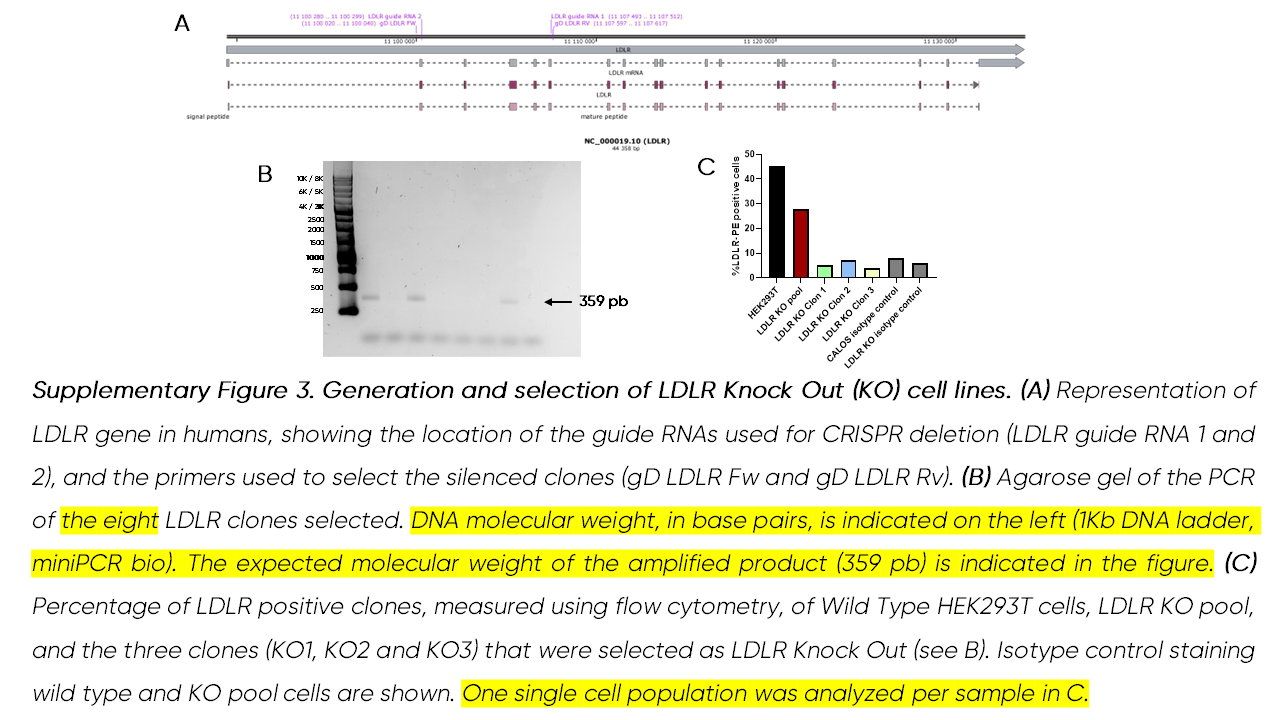

Supplement: Supplementary file 1 [file viruses-16-01216-s001.zip › Figures_Supplementary/fig_supp_3.PNG]

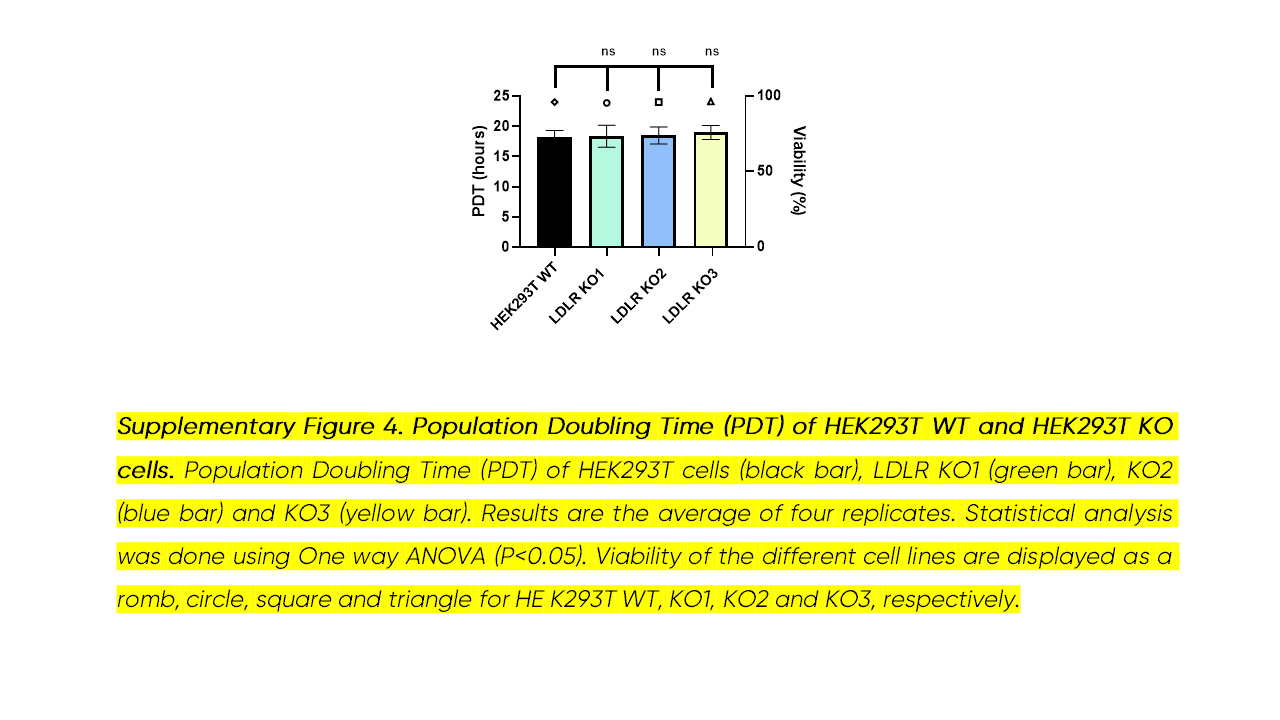

Supplement: Supplementary file 1 [file viruses-16-01216-s001.zip › Figures_Supplementary/fig_supp_4.PNG]

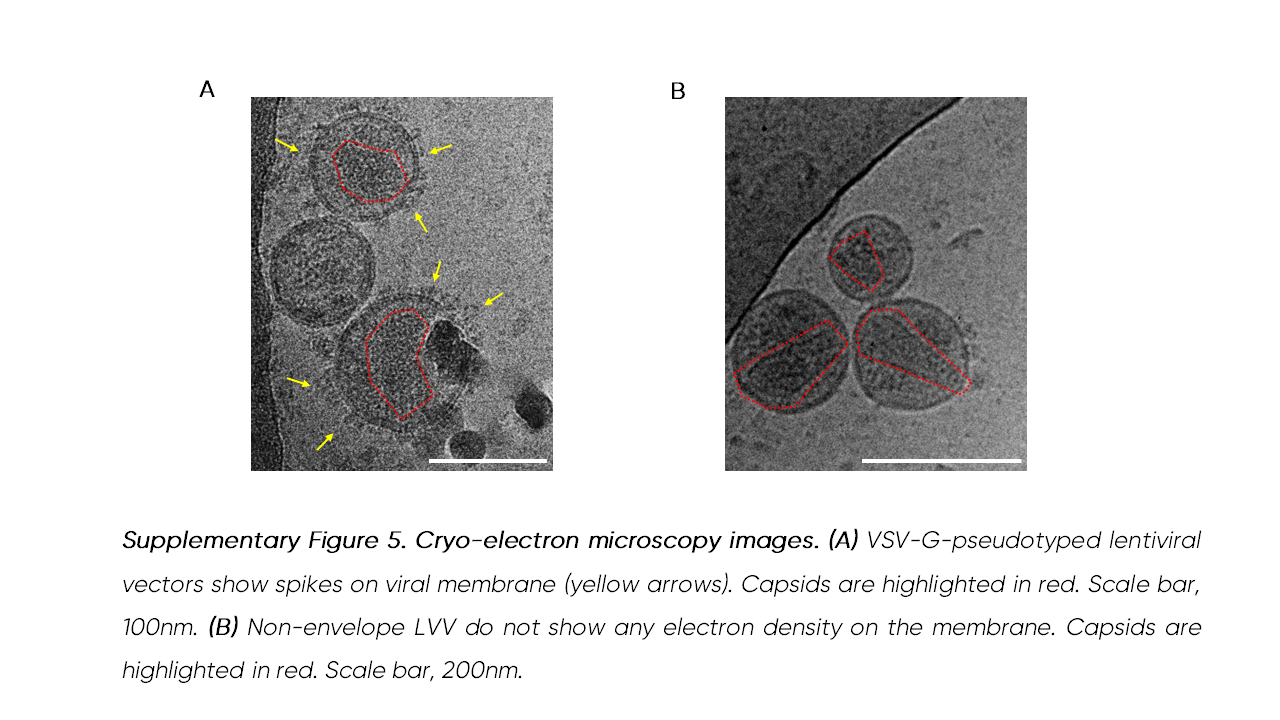

Supplement: Supplementary file 1 [file viruses-16-01216-s001.zip › Figures_Supplementary/fig_supp_5.PNG]

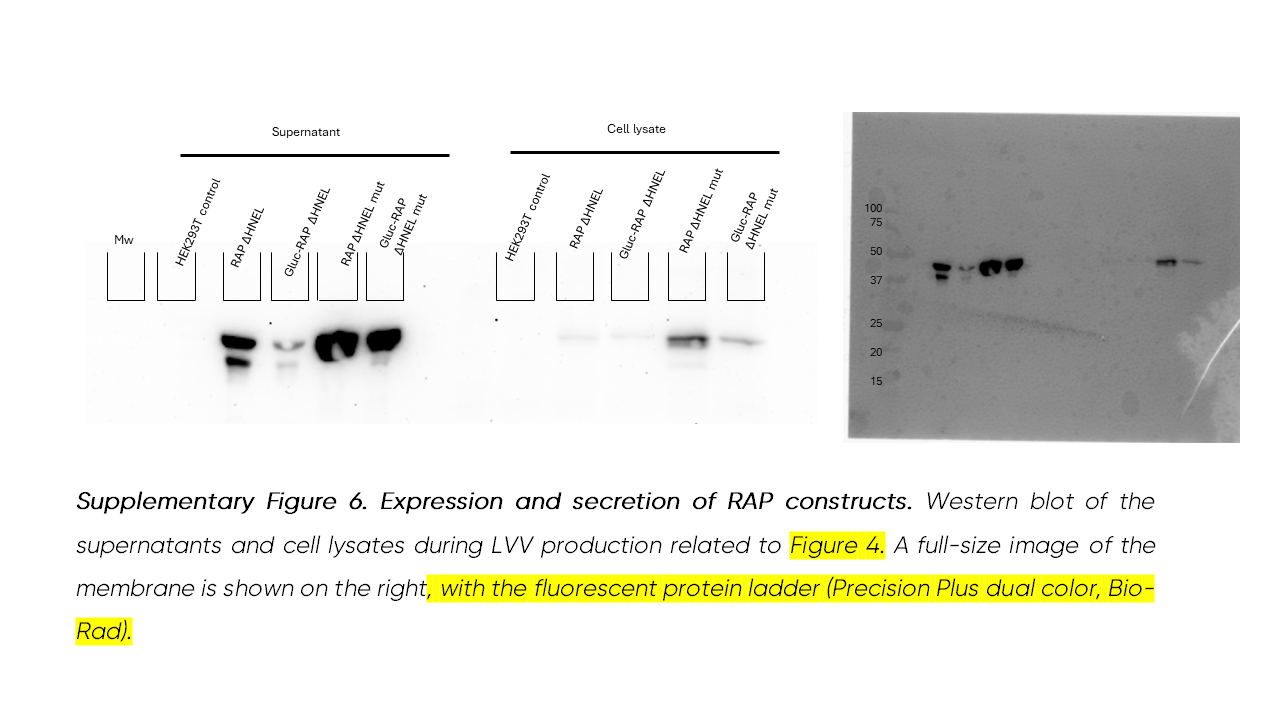

Supplement: Supplementary file 1 [file viruses-16-01216-s001.zip › Figures_Supplementary/fig_supp_6.PNG]
